# Genome-wide identification and expression analyses of *Sm* genes reveal their involvement in early somatic embryogenesis in *Dimocarpus longan* Lour

**DOI:** 10.1371/journal.pone.0230795

**Published:** 2020-04-03

**Authors:** Xue Li, Yan Chen, Shuting Zhang, Liyao Su, Xiaoping Xu, Xiaohui Chen, Zhongxiong Lai, Yuling Lin

**Affiliations:** 1 College of Horticulture, Fujian Agriculture and Forestry University, Fuzhou, Fujian, China; 2 Institute of Horticultural Biotechnology, Fujian Agriculture and Forestry University, Fuzhou, Fujian, China; Wuhan University, CHINA

## Abstract

The Sm proteins are a conserved protein family with Sm motifs. The family includes Sm and Sm-like proteins, which play important roles in pre-mRNA splicing. Most research on the Sm proteins have been conducted in herbaceous plants, and less in woody plants such as *Dimocarpus longan* (longan). And the embryo development status significantly affects the quality and yield of longan. In this study, we conducted a genome-wide analysis of longan *Sm* genes (*DlSm*) to clarify their roles during somatic embryogenesis (SE) and identified 29 *Sm* genes. Phylogenetic analysis deduced longan Sm proteins clustered into 17 phylogenetic groups with the homologous Sm proteins of *Arabidopsis thaliana*. We also analyzed the gene structures, motif compositions, and conserved domains of the longan Sm proteins. The promoter sequences of the *DlSm* genes contained many light, endosperm development, hormone, and temperature response elements, which suggested their possible functions. In the non-embryogenic callus(NEC) and during early SE in longan, the alternative splicing(AS) events of *DlSm* genes indicated that these genes may influence SE development by changing gene structures and sequences. The kinetin(KT) hormone, and blue and white light treatments affected the differentiation and growth of longan embryonic callus(EC) probably by affecting the AS events of *DlSm* genes. Expression profiles showed the possible functional divergence among *Sm* genes, and different hormones and light qualities affected their expression levels. The expression trends of the *DlSm* genes determined by RNA sequencing as fragments per kilobase of exon model per million mapped reads (FPKM) and by real-time quantitative PCR(qRT-PCR) during early SE in longan showed that the expression of the *DlSm* genes was affected by the growth and differentiation of longan SE, and decreased as the somatic embryo differentiation progressed. The results will contributed to understanding the longan *Sm* gene family and provide a basis for future functional validation studies.

## Introduction

Sm family proteins are a family of RNA-binding proteins found in bacteria, archaea, and eukaryotes, including Sm and Sm-like (LSm) proteins[1]. Structurally, Sm proteins have a protein-protein interaction site, the Sm motif, consisting of two parts, Sm1 and Sm2[2, 3]. The Sm motif contains an amino-terminal α-helix and an five-stranded antiparallel β-sheet. The amino-terminal α-helix and the neighboring β1β2β3 sheet fold to form the Sm1 motif, and the β4β5 sheet folds to form the Sm2 motif, and these motifs are involved in the protein–protein interactions that enable the Sm proteins to assemble into RNA-interacting heptameric ring complexes [4, 5]. The Sm core proteins include mainly SmB, SmB′, SmN, SmD1, SmD2, SmD3, SmE, SmF and SmG, which stably bind with U1, U2, U4 and U5 small nuclear RNAs(snRNAs) and other proteins to form particles called small nuclear ribonucleoproteins or snRNPs in the nucleus of cells[6, 7]. These snRNPs can form a larger complex, the spliceosome, which can cleave introns not encoded by the pre-mRNAs, splicing the exons together, and then undergoing a series of modifications to become mRNAs[6], and the mRNAs are transported through the nuclear pores into the cytosol and eventually translated into proteins by ribosomes eventually [6].The central component of the spliceosome, U6 snRNA, is very specific, it binds only to the LSm proteins and not to Sm proteins[7, 8]. The LSm proteins belong to the protein family that has a conserved Sm-like motif and are homologous with the Sm proteins. The LSm proteins comprises mainly nine proteins (LSm1-9), most of which (LSm1-8) are involved in forming heptameric ring complexes. LSm1–7 form a cytoplasmic complex that can bind to oligoadenylated mRNA and promote mRNA capping. LSm2–8 form a complex that can stabilize U6 snRNA and play a role in pre-mRNA splicing. LSm2–7 have been shown to be closely related to their Sm counterparts, SmD1 to SmG, whereas LSm1 and LSm8 may be closely related to the SmB subfamily [8–10]. Larger proteins that not only have an LSm domain but also other protein domains, (e.g., LSm10-16, ataxin-2, and archaeal Sm3) have been found recently[11].

Until now, most of the studies on the Sm protein family have focused mainly on protein structure and splicing principle, and mainly in bacteria, yeast, and humans. Stark et al. [12] identified seven LSm proteins (LSm1–7) in red algae, indicating that red algae do not contain LSm8. Subsequent studies by Reimer et al. [13] demonstrated that the LSm1-7 complex was involved in splicing and cytoplasmic mRNA degradation. In higher plants, the Sm protein family has been identified only in *Arabidopsis*, rice, and maize. The genetic structure, phylogeny, chromosomal location, selection stress and expression analysis of the *Sm* gene family have been reported in *Arabidopsis*[14]. In rice and corn, the systematic development, chromosome mapping, gene organization, adaptive evolution, expression profile, and functional network of the *Sm* gene family have been studied[14, 15]. The functions of some of the *Arabidopsis* Sm proteins have been verified. For example, the expression of LSm5 was found to regulate the circadian clock and the mutation of *LSM5* or *LSM4* prolonged the circadian rhythm in *Arabidopsis*[16]. The function of the LSm2–8 complex in pre-mRNA splices is controlled by external signals, which differentially regulated the adaptability of *Arabidopsis* to abiotic stress[17]. Further, overexpression of the *SAD1* gene, which encodes the LSm5 protein enhanced the salt tolerance of *Arabidopsis*[18]. Thus, current knowledge about the Sm proteins indicates they can promote snRNA cap modification and target snRNPs to the appropriate cell locations, but it is unclear whether they have other functions. Until now, most of the research on the Sm protein family has been conducted in herbaceous plants, so not much is known about their roles in woody plants, especially in *Dimocarpus longan* (longan).

Longan is an important tropical and subtropical woody fruit tree in China. The fruit is highly nutritious and the embryogenesis status of the plant significantly affects fruit quality and yield. Cell biology and molecular biology studies of somatic embryogenesis (SE) in longan have greatly progressed the understanding of the SE process. A large number of molecular and gene regulation mechanisms inherent in the development of longan SE have been studied in our laboratory. Until now, the focus has been on the regulation of microRNAs[19, 20], regulation of DNA methylation-related genes[21, 22], secondary metabolism [23, 24], and stress[25, 26], as well as genes related to the regulation of growth and development[27–30] related genes in longan SE. However no studies have investigated alternative splicing factors associated with longan SE, and the function of most plant Sm proteins is still unclear.

In this study, we conducted a the genome-wide analysis, alternative splicing(AS) analysis, and investigated the expression partterns of *Sm* genes in longan. Bioinformatics analysis of the longan *Sm* family was carried out, and 29 *Sm* genes were identified. The physicochemical properties, conserved motifs and protein domains and phylogenetic tree of the Sm protein sequences, and the *cis*-acting elements in the promoters of the *Sm* genes were analyzed. The AS events of the *Sm* genes in the non-embryogenic callus(NEC) stage and the early SE, longan embryonic callus(EC) stages, were also analyzed under different hormone and light quality treatments. Finally, the FPKM values of the *Sm* genes in NEC, early SE stages and different longan tissues were also analyzed. The expression of *DlSm* genes in longan SE stages were verified by real-time quantitative PCR(qRT-PCR). The results of this study will provide a basic resource for future research on the function of the longan *Sm* gene family.

## Materials and methods

### Plant materials

The original experimental material was 'Honghezi' longan EC, which was provided by the Institute of Horticultural Biotechnology, Fujian Agriculture and Forestry University[31]. We used the culture method of Lai Zhongxiong as reference to obtain the longan EC, incomplete embryogenic compact structure(ICpEC), globular embryo(GE) and NEC by adding different concentrations of 2,4-Dichlorophenoxyacetic acid(2,4-D) to the culture medium[32, 33]. The original experimental materials used in qRT-PCR and the original experimental materials used in transcriptome sequencing were taken from different single plant of 'Honghezi' cultivar. This study did not involve any near threatened species and did not require a statement of ethical approval.

### Identification of *DlSm* gene family members

We retrieved *Arabidopsis* Sm protein sequences[15] and then used the *Arabidopsis* genomic Sm amino acid sequence as a probe sequence and local blasting with the longan genome database(NCBI accession number: BioProject PRJNA305337)[34]. 29 longan *Sm* genes were obtained by screening annotations. According to the genome annotation, we used the NCBIBLAST homologous alignment analysis to compare the genomic DNA(gDNA) sequences, coding sequences(CDS), and amino acid sequences of *Sm* with DNAMAN6.0 software. According to the annotations of the *Sm* family of *Arabidopsis thaliana* in the longan genome and their chromosomal location, the longan *Sm* genes were named and identified by reference to the common nomenclature of most species in the NCBIBLAST homologous alignment. Among them, *DlLSm14b*, *DlSmD3* and *DlLSm7b* had no complete amino acid sequences, and subsequent analysis of protein physicochemical properties, gene structure, conserved motif, and protein domain will not be carried out.

### Analysis of the phylogenetic tree and the conservative domain of the DlSm protein family system

The amino acid sequences of longan and *Arabidopsis Sm* genes were compared by DNAMAN software. Then based on neighbor-joining(NJ) method, 61 *Sm* genes amino acids from longan and *Arabidopsis thaliana* were constructed by MEGA7 software. Next, the sequences with large homology differences were manually deleted and 1000 boot strap samples were carried out. Combined with CDD(Conserved Domain Database)(https://www.ncbi.nlm.nih.gov/Structure/cdd/wrpsb.cgi), the conserved domains of protein sequences in longan and *Arabidopsis thaliana* were analyzed by ITOL (https://itol.embl.de/).

### *DlSm* gene structure and conservative domain analysis

We analyzed the introns and exons of 61 *Sm* genes in longan and *Arabidopsis thaliana* by GSDS2.0 (http://gsds.cbi.pku.edu.cn/). We used ExPASy (https://www.expasy.org/) to predict the basic physical and chemical properties of 26 DlSm amino acid sequences, including the number of amino acids(aa), molecular weight (Mw), and other electric point (pI), N-terminal signal peptides. After MEME online analysis (http://meme-suite.org/) of conservative motif of longan Sm proteins, tbtools was used to draw the obtained motif.

### Analysis of *cis*-acting elements of *DlSm* genes promoter

We extracted the promoter sequences of 3000bp upstream of ATG in *DlSm* genes. PlantCARE online website was used to predict its *cis*-acting elements, in which the *DlLSm1a* promoter sequence contained many N, and the gene promoter was not analyzed for the time being. The different types of *cis*-acting elements of each gene promoter were counted and plotted by Excel software.

### Analysis of AS of *DlSm* gene family

The longan transcriptome datasets were used to analyze the AS events of *DlSm* gene family in longan NEC and the early stage of SE(EC, ICpEC and GE)(NCBI SRA accession: PRJNA565345)[35]. The datasets of longan EC treated with different hormones and longan EC under two different light-quality treatments(blue and white light) have not been published. The hormone treatments were as follows: 2,4-D&KT: Murashige and Skoog(MS) medium+ 2,4-D(1.0 mg/L)+ KT(0.5 mg/L), 2,4-D: MS medium+ 2,4-D(1.0 mg/L), KT: MS medium+ KT(0.5 mg/L) and MS medium without any hormones. The materials were dark cultured in these liquid medium for 24 hours.The datasets of light quality treatment have been published, and the specific processing methods refer to the study of Li et al.[36]. Adding 20 g· L^-1^ sucrose, 6 g· L^-1^ agar and 4.5μM 2,4-D to MS medium for light treatment experiments of longan EC. The starting material for each bottle was 0.04 g. Longan ECs were incubated in a plant light chamber for 25 days and darkly treated as a control. The intensity and period of blue light (457 nm) and white light were fixed at 32μmol· m^−2^·s^−1^and 12 h·Day^−1^, respectively[36]. All experimental materials were stored at -80°C for subsequent analysis. We used rMATS[37] to detect the AS events between samples. AS events under different developmental stages and different hormone treatments of longan EC were collected from more than three samples among every dataset. Under different light quality treatments, the AS events were collected from more than two samples among every dataset.

### Analysis of specific expression of *DlSm* family genes

We introduced the FPKM value of nine organs(flower bud, flower, leaf, pericarp, pulp, root, seed, stem, young fruit) of 'Sijimi' longan cultivar (the datasets was deposited at NCBI under GSE84467) and NEC, EC, ICpEC, GE of 'Honghezi' longan cultivar [34, 35], and longan EC treated with different hormones(the datasets have not been published) and light quality[36] to the Omicshare online website to map the heat expression of gene expression (http://www.omicshare.com/tools/index.php/). The disclosure of the transcriptome datasets is the same as above.

### Real-time quantitative PCR

The total RNA of longan was extracted by TransZol Up Kit and complementary DNA(cDNA) was synthesized by PrimerScript RT Reagent Kit (TaKaRa). The specific primers for *DlSm* genes were designed by dnaman software. qRT-PCR refers to the method of Lin et al.[38], and we used eukaryotic elongation factor 1-alpha (*EF-1α*) as internal reference gene. We amplified qRT-PCR on a Roche LightCycler 480 instrument (Roche Applied Science, Switzerland). The qRT-PCR reaction system (20 μl) consists of 10 μL SYBR premix ex TaqTM II (TaKaRa), 7.4 μL ddH_2_O, 0.8 μL upstream and downstream specific primers (S1 Table) and 1 μL cDNA template. The reaction procedure is 95°C pre-denaturation 30 s, 95°C denaturation 5 s, 60°C annealing 30 s, 72°C extension 15 s, 40 cycles. We used the 2^−ΔΔCt^ method to calculate expression values and then used Graphpad prism 7.0 to analyze the data and make the related image.

## Results

### Identification of *DlSm* gene family members

To identify members of the longan *Sm* gene(*DlSm*) family, the amino acid sequences of *Arabidopsis* Sm proteins were listed as probe sequences, and we searched the sequences by using local blast and NCBIBLAST. The initial screening identified 30 potential members. We found that only one base in the coding sequences of Dlo_022245.1 and Dlo_032946.1(LSm6) was different, the translated amino acid sequences were exactly the same, and the translated start sites were also the same, so we regarded them as the same sequence, and used the Dlo_032946.1 for further analysis. Therefore, 29 *Sm* genes were identified in the longan genome. The longan *Sm* genes were named on the basis of their conserved domains, chromosomal locations, and annotations of the homologous *Sm* genes in *Arabidopsis thaliana*, as *DlLSm1*-*7*, *DlLSm14a*~*DlLSm14e*, *DlLSmD1*, *DlSmB*, *DlSmD*, *DlSmD1a*, *DlSmD1b*, *DlSmD2a*, *DlSmD2b*, *DlSmD3*, *DlSmEa*, *DlSmEb*, *DlSmF*, and *DlSmG* (Table 1). The physicochemical properties of deduced DlSm proteins were analyzed using the ExPASy server. We found the DlSm amino acid sequences were from 74 to 862 amino acids long, their molecular weights were from 8682.79ku to 92952.89ku, and their electric points were from 4.45 to 11.73. None of the 29 Sm sequences had a signal peptide, so they were not secretory proteins.

**Table 1:** Information of longan *Sm* family genes.

Gene ID	Gene name	Comments to members of *Arabidopsis*	Gene localization	aa number	Mw	pI	Signal peptide
Dlo_023124.1	*DlLSm1a*	*AT3G14080 AtLSm1b*	scaffold504:56684:58077	151	17067.94	8.94	No
Dlo_023126.1	*DlLSm1b*	*AT3G14080 AtLSm1b*	scaffold504:65206:66779	125	14179.59	5.26	No
Dlo_024845.1	*DlLSm2*	*AT1G03330 AtLSm2*	scaffold556:163264:167575	377	42539.65	9.16	No
Dlo_009273.1	*DlLSm3*	*AT1G76860 AtLSm3b*	scaffold185:484650:485838	74	8682.79	4.45	No
Dlo_014195.1	*DlLSm4a*	*AT5G27720 AtLSm4*	scaffold263:327344:329240	146	15843.89	10.01	No
Dlo_021351.1	*DlLSm4b*	*AT5G27720 AtLSm4*	scaffold449:612750:615216	150	16135.20	10.14	No
Dlo_031761.1	*DlLSm4c*	*AT5G27720 AtLSm4*	scaffold845:311468:313948	150	16135.20	10.14	No
Dlo_023673.1	*DlLSm5a*	*AT5G48870 AtLSm5*	scaffold52:850634:851892	120	13777.51	5.54	No
Dlo_012057.1	*DlLSm5b*	*AT5G48870 AtLSm5*	scaffold224:406385:407477	87	9599.07	4.50	No
Dlo_032946.1	*DlLSm6*	*AT2G43810 AtLSm6b*	scaffold908:10301:13180	91	9794.13	9.13	No
Dlo_028712.1	*DlLSm7a*	*AT2G03870 AtLSm7*	Scaffold70:1068910:1071695	99	10746.27	4.79	No
Dlo_034364.1	*DlLSm7b*	*AT2G03870 AtLSm7*	Scaffold1018:97362:100063	No termination codon			No
Dlo_036673.1	*DlLSm8*	*AT1G65700 AtLSm8*	Scaffold4:715280:716076	99	10759.14	4.68	No
Dlo_026784.1	*DlLSm14a*	*AT1G26110 AtDCP5/*(*LSm14g*)	scaffold62:1097537:1107145	596	63092.69	6.19	No
Dlo_038405.1	*DlLSm14b*	*AT1G26110 AtDCP5/*(*LSm14g*)	scaffold62:1151845:1157828	No termination codon			No
Dlo_006168.1	*DlLSm14c*	*AT1G26110 AtDCP5/*(*LSm14g*)	Scaffold145:358361:366094	862	92952.89	9.18	No
Dlo_011575.1	*DlLSm14d*	*AT1G26110 AtDCP5/*(*LSm14g*)	scaffold217:3535:10764	627	67457.47	6.27	No
Dlo_029021.1	*DlLSm14e*	*AT5G45330 AtDCP5-L/*(*LSm14f*)	Scaffold711:39163:43435	568	61111.37	8.62	No
Dlo_020209.1	*DlLSmD1*	*AT4G18372/*(*AtLSmD1*)	scaffold41:74616:74960	99	10876.35	8.61	No
Dlo_033912.1	*DlSmB*	*AT4G20440 AtSmBb*	scaffold98:392671:393516	281	29663.61	11.28	No
Dlo_024823.1	*DlSmD1a*	*AT4G02840 AtSmD1b*	scaffold556:554:6323	224	24725.29	10.24	No
Dlo_031697.1	*DlSmD1b*	*AT4G02840 AtSmD1b*	scaffold842:61514:61871	80	8856.32	11.73	No
Dlo_014464.1	*DlSmD2a*	*AT3G62840 AtSmD2b*	scaffold27:468436:469034	104	12077.18	9.74	No
Dlo_009060.1	*DlSmD2b*	*AT3G62840 AtSmD2b*	scaffold1806:24804:26735	105	12063.15	9.74	No
Dlo_038407.1	*DlSmD3*	*AT1G20580 AtSmD3b*	scaffold69:954276:957329	No termination codon			No
Dlo_015839.1	*DlSmEa*	*AT2G18740 AtSmEb*	Scaffold3:469665:469951	79	9194.89	9.89	No
Dlo_019122.2	*DlSmEb*	*AT2G18740 AtSmEb*	Scaffold38:1862876:1865058	79	9167.82	9.82	No
Dlo_021780.1	*DlSmF*	*AT4G30220 AtSmF(AtSmFa)*	scaffold46:632326:633944	88	9910.28	4.43	No
Dlo_009662.1	*DlSmG*	*AT2G23930 AtSmGa*	scaffold19:1245388:1247149	80	8906.33	9.22	No

For the convenience of drawing, the name of *Arabidopsis thaliana* gene in parentheses is named according to its protein domain and amino acid size, which is only applicable in this paper.

### Evolutionary relationship of the longan and *Arabidopsis* Sm proteins family and analysis of the protein domains

**Fig 1:**
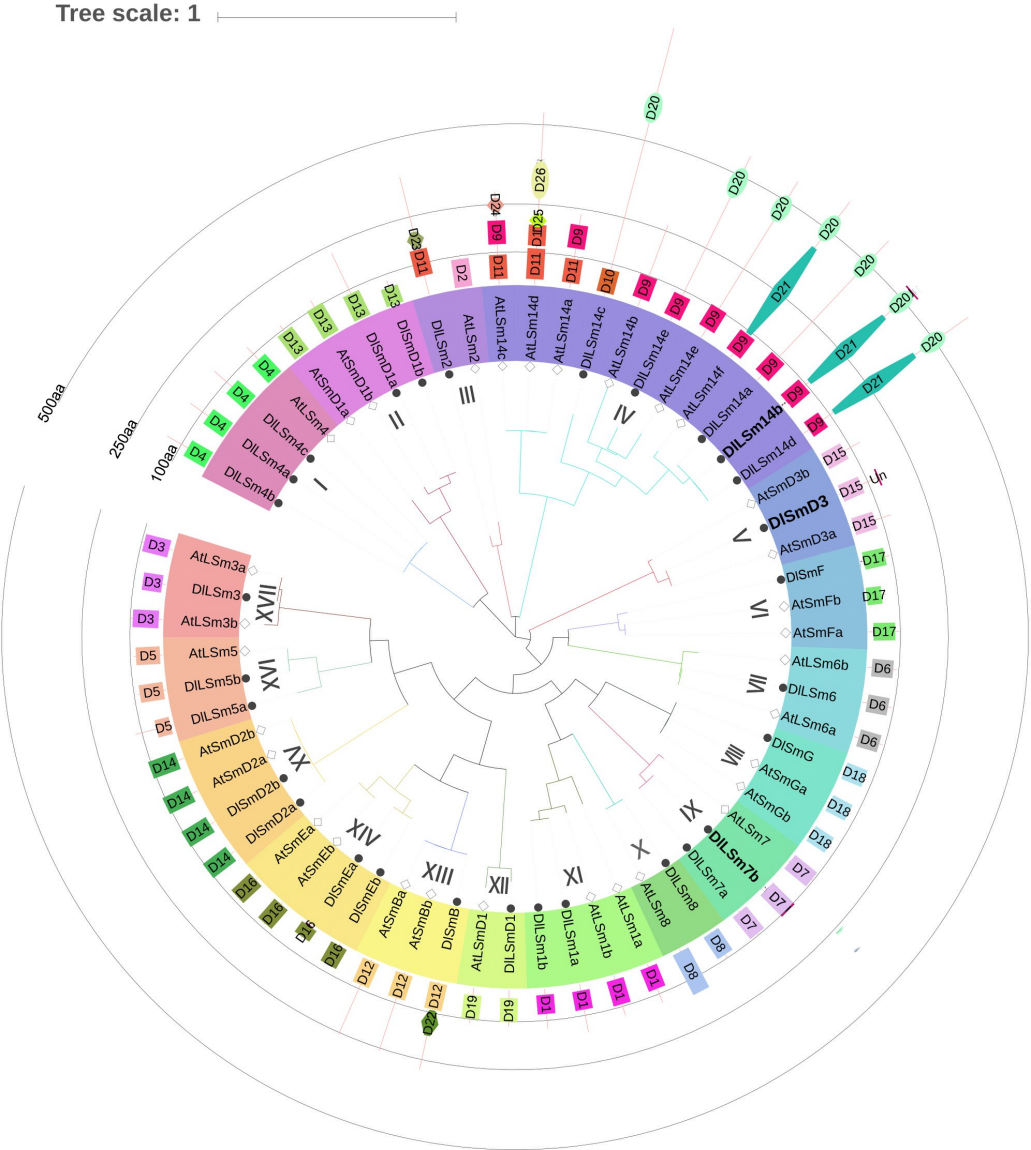
Longan and *Arabidopsis* Sm protein family evolutionary tree with their protein domains. D1~D26 are LSm1, LSm2, LSm3, LSm4, LSm5, LSm6, LSm7, LSm8, LSm14-N, LSm14, LSm super family, SmB, SmD1, SmD2, SmD3, SmE, SmF, SmG, LSmD1, FDF, Atrophin -1, DNA-Pol3, HMA, Kelch1, F-box, BTB protein domains; labeled the CDS sequence of DlLSm14b, DlSmD3, DlLSm7b without termination codon.

To examine the phylogenetic relationship of the Sm proteins between *Arabidopsis thaliana* and longan, we used MEGA7 to construct an evolutionary tree based on their amino acid sequences. We found the Sm proteins clustered into 17 groups I-XVII (Fig 1), and each group contained the Sm proteins of *Arabidopsis* and longan. Group IV contained 11 LSm14 proteins and was the largest group. Among them, the *Arabidopsis* proteins AtLSm14a, AtLSm14c, and AtLSm14d in Group IV shared a closer relationships than other members of this group. There were five DlLSm14 proteins in Group IV, and was also the largest branch of DlSm phylogeny, and one DlSm protein in each of nine groups.

Analysis of the protein domains in the longan and *Arabidopsis* of Sm proteins using the CDD and InterPro revealed that they all contained the Sm-like super family domain(Fig 1). The DlLSm2 protein contained the LSm superfamily domain and an HMA domain, whereas the AtLSm2 protein only contained only the LSm2 protein domain. The DlLSm14 proteins contained an FDF domain in addition to the LSm superfamily domain. The DlLSm14b and DlLSm14d proteins also contained an Atrophin-1 super family domain. The DLSmB protein contained the LSm superfamily domain and a DNA_pol3_delta2 superfamily domain. AtLSm14a, AtLSm14c, and AtLSm14d, which formed a cluster separate from the other members of Group IV, contained protein domains that were quite different from the domainsthose of the other members. Except for the Sm proteins in Groups III and Group IV, the proteins of other groups contained the same LSm superfamily domain, which suggested the functions of the Sm proteins in these two groups members may had changed during evolution. The Sm protein sequences were generally short, and the characteristic LSm domain was located between positions 1 and 150aa, indicating that the domain was relatively conservatived.

### *DlLSm* gene structure and conserved motif analysis

**Fig 2:**
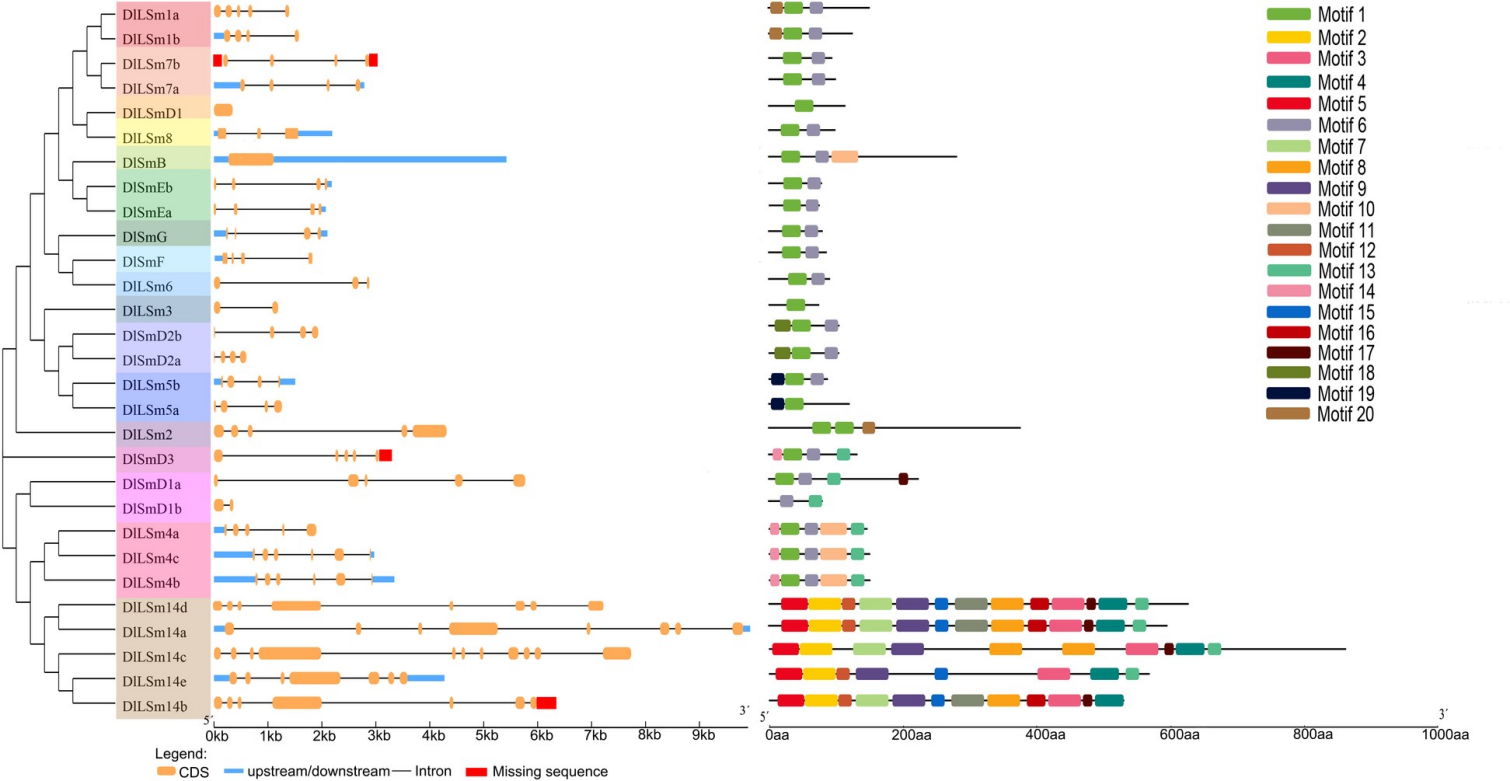
Structural analysis of *DlSm* genes and their encoded proteins. The unknown sequences were located in the non-coding region or the non-conservative region, which did not affect the analysis results.

Introns are an important part of eukaryotic genes and play key roles in genetic evolution. To investigate the mechanism of structural evolution of the *Sm* genes, we compared the exon–intron structures of the longan *Sm* genes (Fig 2). The results of GSDS analysis showed that the number of introns in the *DlSm* genes contained from zero to ten introns, with most of the genes having three to four introns, which was similar to what was found for the *Arabidopsis Sm* genes. The *Sm* genes that encoded members of Group IV contained six to ten introns, whereas the genes that encoded members of the other groups had one to five introns, which indicated they were quite different. In addition, the genes that encoded the members of each clustered usually had similar genetic structures and there were also large differences in the structures of the genes among the different groups. The genes encoding members of Groups XII and XIII had no introns. Overall, the numbers and locations of introns in the *Sm* genes varied widely among the different groups, probably because of differences in the intron gain and loss rates[39]. Besides, the differences in the lengths and structures of the longan *Sm* genes, the quantity and distribution of the exons and introns were different among the different groups, which may lead to differences in the differentiation of gene function.

A total of 20 different motifs were identified in the longan Sm proteins by MEME(Fig 2). The most closely related Sm proteins in the phylogenetic tree had similar motif compositions, reflecting the functional similarity between them. Of the 29 DlSm proteins, only DlSmD1b and members of Group IV members did not contain Motif 1, and 20 of all members contained Motif 6. The motif composition of the proteins in Group IV was very different from that of proteins in the other groups, which confirmed that Group IV was far away from the other groups. Together, these results provide a reference for the functional diversity of the longan Sm proteins.

### Analysis of *cis*-acting elements in the promoters of *DlSm* genes

**Fig 3:**
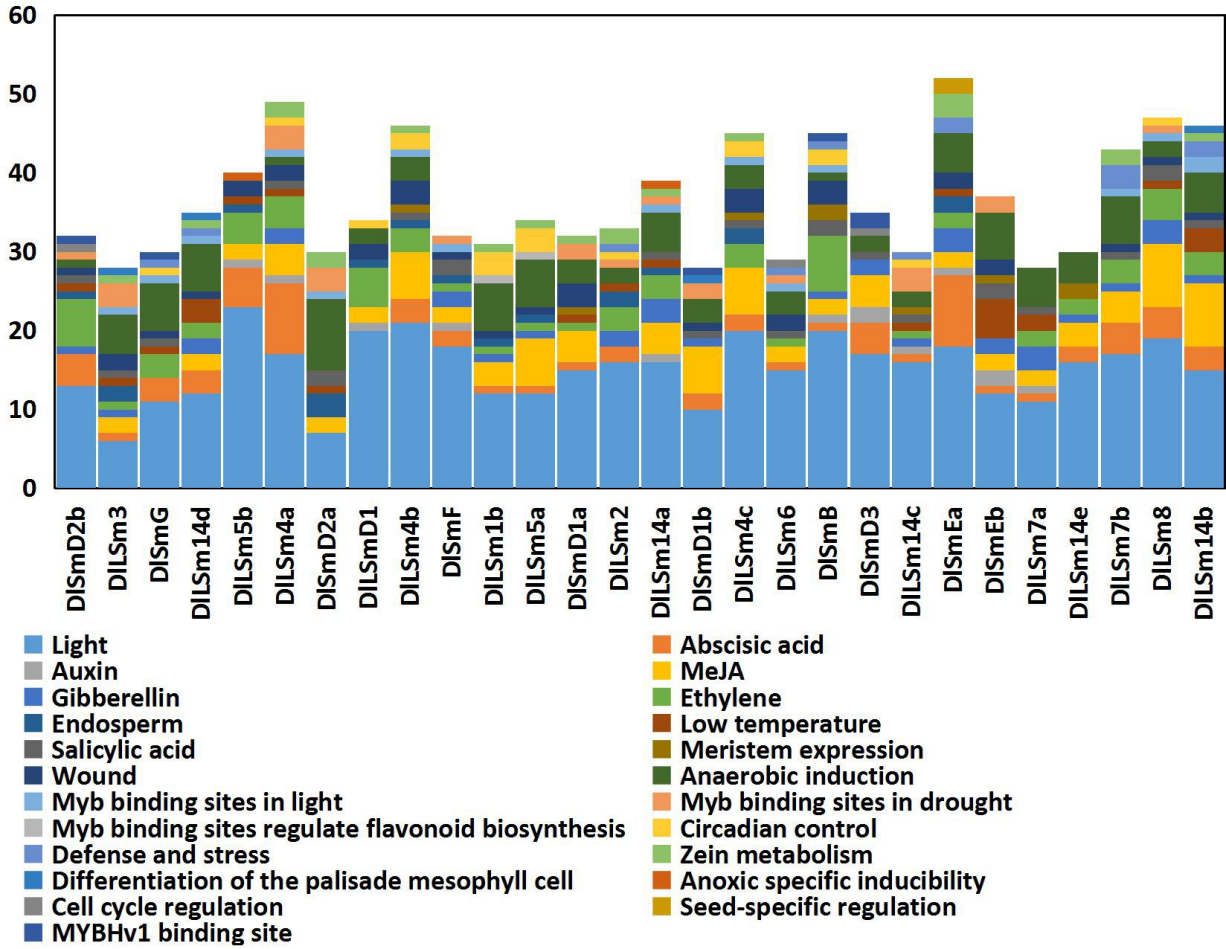
The distribution of *cis*-acting elements of the *DlSm* promoters. The 3.0 kb regulatory region upstream of ATG were analyzed by the PlantCARE software.

We used PlantCARE to analyze the promoters of 28 longan *Sm* genes and the results were shown in Fig 3. The promoters of all 28 genes contained the basic components such as CAAT-box and TATA-box. The identified *cis*-acting elements in these promoters were roughly classified into 5 categories: plant hormone response elements, stress response elements, tissue-specific related action elements, photoresponsive elements, and cell cycle response elements. The longan *Sm* genes contained a large number of photoresponsive elements, and 93% of the promoters sequences contained a large number of anaerobic induction response elements. More than 80% of the promoters contained abscisic acid(ABA) and methyl jasmonate(MeJA) response elements, about 75% of the members responded to wound, and 50% of the genes contained photoreactive MYB binding sites and protein metabolism regulatory elements. The *Sm* promoters also contained elements that responded to physiological circadian rhythm control and endosperm development. Among the 28 *Sm* genes, the *DlLSm1b* and *DlLSm5a* promoters contained MYB transcription factor action sites that regulate flavonoid biosynthesis; the *DlLSm4c*, *DlSmEa*, *DlSmD2b* promoters respond to cell cycle regulation; and the *DlSmEa* promoter responds to seed-specific regulation. Seven of the *Sm* promoters (*DlLSm4b*, *DlLSm4c*, *DlSmD1a*, *DlSmB*, *DlSmEb*, *DlLSm14c*, *DlLSm14e*) contained *cis*-acting elements associated with meristem expression. These results can provide a basis for the regulatory network of longan S*m* genes.

### AS analysis of *DlSm* family in the NEC and early SE in longan

**Fig 4:**
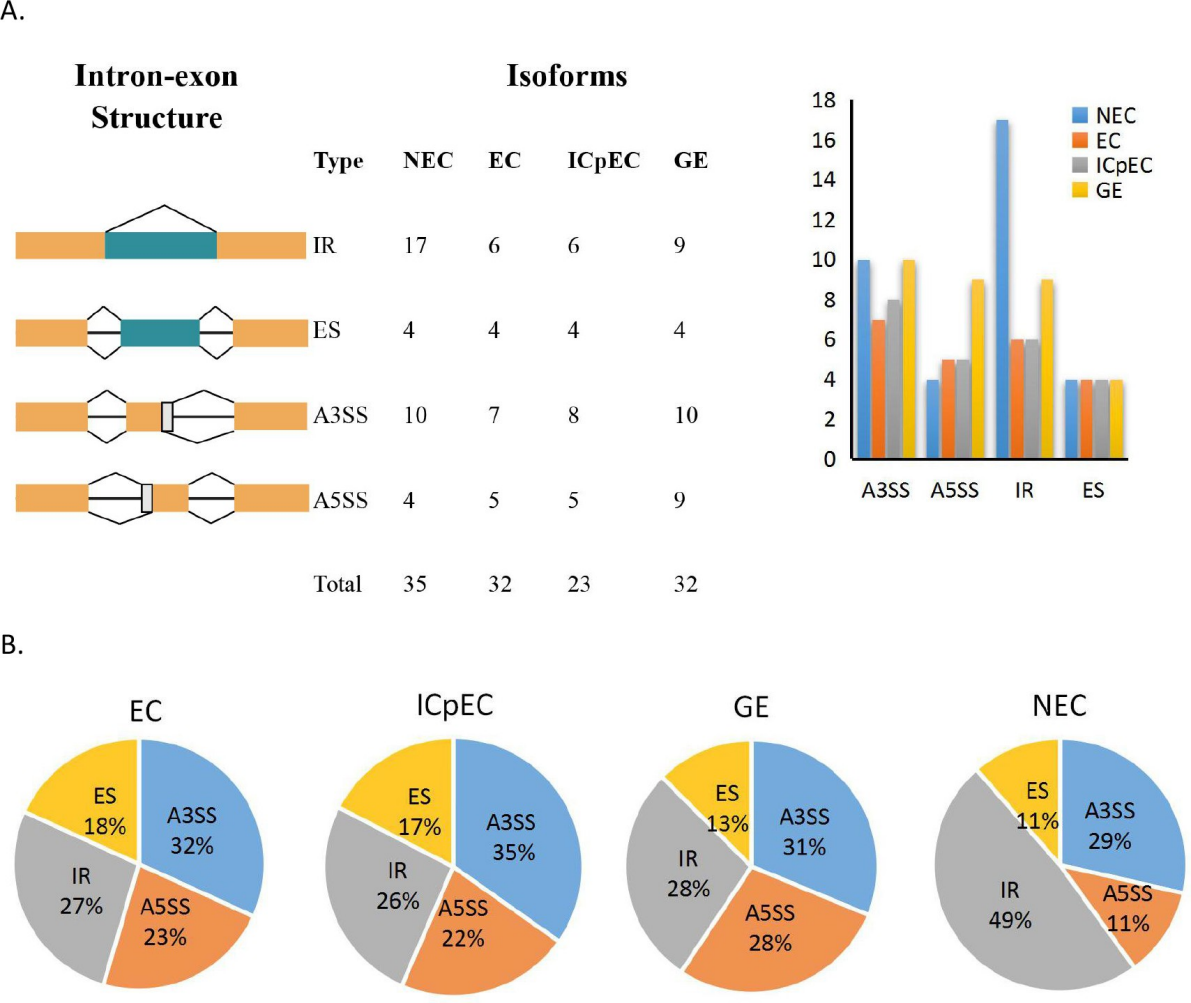
Distribution of AS events in NEC and early SE stages of longan. **A** four splicing event patterns and their number of events in longan early SE. **B** distribution of AS events in longan NEC, EC, ICpEC, GE stages.

We used our group’s transcriptional databases and screened for four different types of splicing events in longan NEC and in three stages of early SE (EC, ICpEC and GE), namely alternative 3′ splice site(A3SS) and alternative 5′ splice site(A5SS), intron retention (IR), and exon skipping (ES). The results showed that, the number of AS events was more in the NEC(35) compared with the numbers in the other stages, and the number of AS events was lowest in the ICpEC(23) stage (Fig 4A). The number of IR events in the NEC stage was significantly higher than the numbers in the other three stages. There were up to nine A5SS events in GE stage, and the numbers of ES events were the same in all four stages. The results showed that the percentage of IR events was highest in NEC stage (49%) and IR events accounted for the largest proportion of AS events in the NEC stage (Fig 4B). There were no obvious differences in the percentages of AS events in EC, ICpEC and GE stages.

**Fig 5:**
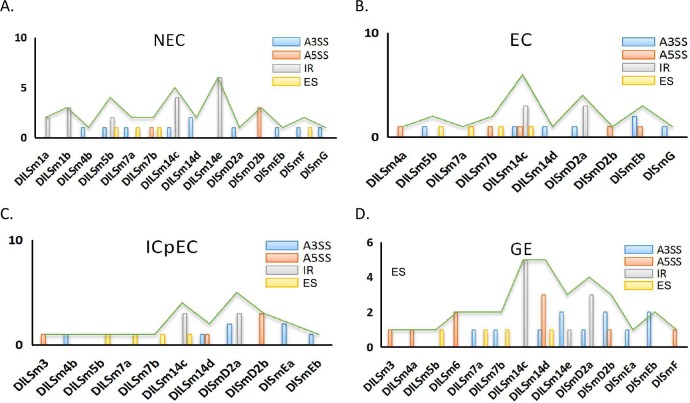
Distribution of four types of AS events in the *DlSm* family of longan NEC and early SE stages.

We also compared the distribution of AS events among the *DlSm* genes in longan NEC and early SE stages(Fig 5; S2 Table). The A3SS events had the highest frequency, occurring in nine, six, five and eight genes at the NEC, EC, ICpEC, and GE stages, respectively. In the NEC stage, 14 genes showed AS, and only three genes had more than two types of AS events(Fig 5A), among them, *DlLSm14c* had four types of AS events occurred in *DlLSm14c*. Only one type of AS events occurred in the other 11 genes(Fig 5A). Five genes had IR events, but the IR events in *DlLSm1a* and *DlLSm1b* occurred only in the NEC stage, where the number of IR events per gene was significantly higher than the numbers of the other types of AS events. The total number of IR events per genes also was the highest in the NECs and was higher than the other three AS types(Fig 5A). In the other three stages, although the number of IR events per gene was significantly higher than that of other types of AS events, the overall frequency of A3SS events was higher than that of IR events. The AS events in *DlLSm1a* and *DlLSm1b* occurred only in the NEC stage and in *DlLSm6* they occurred only in the GE stage, whereas in *DlLSm14c*, *DlLSm14d*, *DlLSm7a*, *DlLSm7b*, *DlSmd2a*, and *DlSmEb* AS events occurred in all four stages(Fig 5). The AS events of *DlLSm14e* were the highest in the NEC stage, and that of *DlSmD2a* were the highest in the ICpEC stage(Fig 5A and 5C). AS events of *DlLSm14* were highest in the EC and GE stages(Fig 5B and 5D). The types of AS events types in *DlLSm14c* decreased gradually as SE progressed; that is, four, two and one types of AS events occurred in the EC, ICpEC and GE stages, respectively(Fig 5B–5D). Our study indicated that a large number of AS events detected in the NEC stage may affect SE in longan in particular, the high occurrence of IR events. The observed decrease in the types of AS events in *DlLSm14c* as SE progressed may promote the differentiation of longan EC stage into GE stage.

### AS analysis of *DlSm* family in longan EC stage treated with different hormones

**Fig 6:**
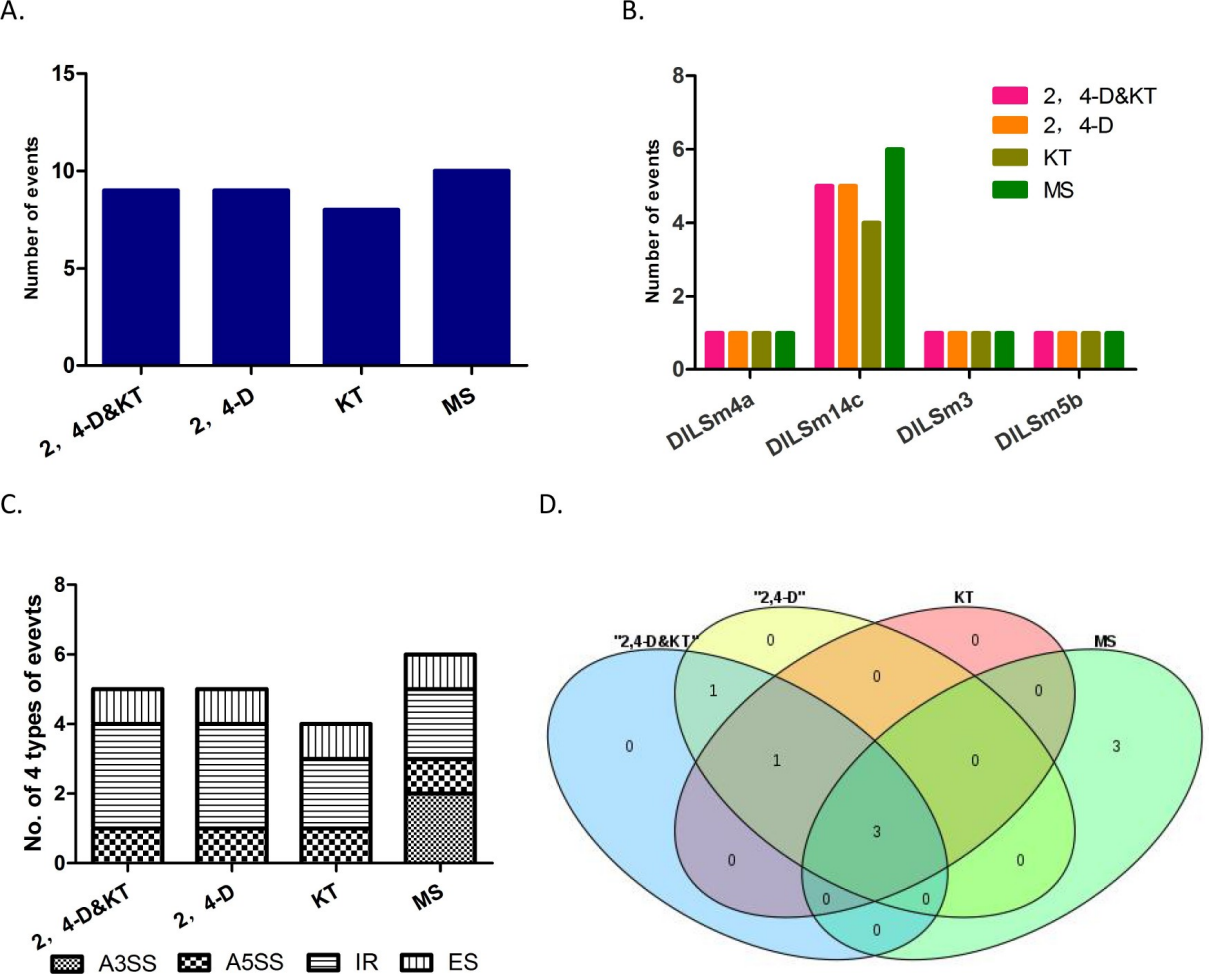
AS events of *DlSm* genes in longan EC stage treated with different hormones. **A** Quantities of AS events in *DlSm* family under different hormone treatments. **B** Distribution of AS events in *DlSm* family under different hormone treatments. **C** The quantity and distribution of four types of AS events in *DlLSm14c* under different hormone treatments. **D** Specific splicing of *DlLSm14c* under different hormone treatments.

2,4-D and KT play important roles in the growth and differentiation of longan EC[31, 40, 41]. To further explore the effects of different hormones on AS events of *DlSm* genes, we used our group’s transcription data to analyze the AS events of longan EC treated with 2,4-D&KT, 2,4-D, KT and MS medium as control. All three hormone treatments reduced the numbers of the AS events in the *DlSm* genes compared with numbers in the control (Fig 6A). AS events occurred in four genes *DlLSm4a*, *DlLSm3*, *DlLSm5b*, *DlLSm14c* (Fig 6B; S3 Table), and were highest in *DlLSm14c*. Further the numbers of AS events that occurred under each on the hormone treatments were the same in all the genes, except *DlLSm14c*, in which the numbers of AS events were different under the different treatments. Under the 2,4-D&KT and 2,4-D treatments, the IR events of *DlLSm14c* increased, and A3SS events occurred only in the control (Fig 6C). In addition, two, two, and one specific AS events occurred in *DlLSm14c* under the 2,4-D&KT, 2,4-D and KT treatments, respectively, compared with the control (Fig 6D). These results suggested that 2,4-D and KT may affect the growth and differentiation of longan EC by affecting the AS of *DlLSm14c*.

### AS analysis of the *DlSm* family in longan EC treated with different light qualities

**Fig 7:**
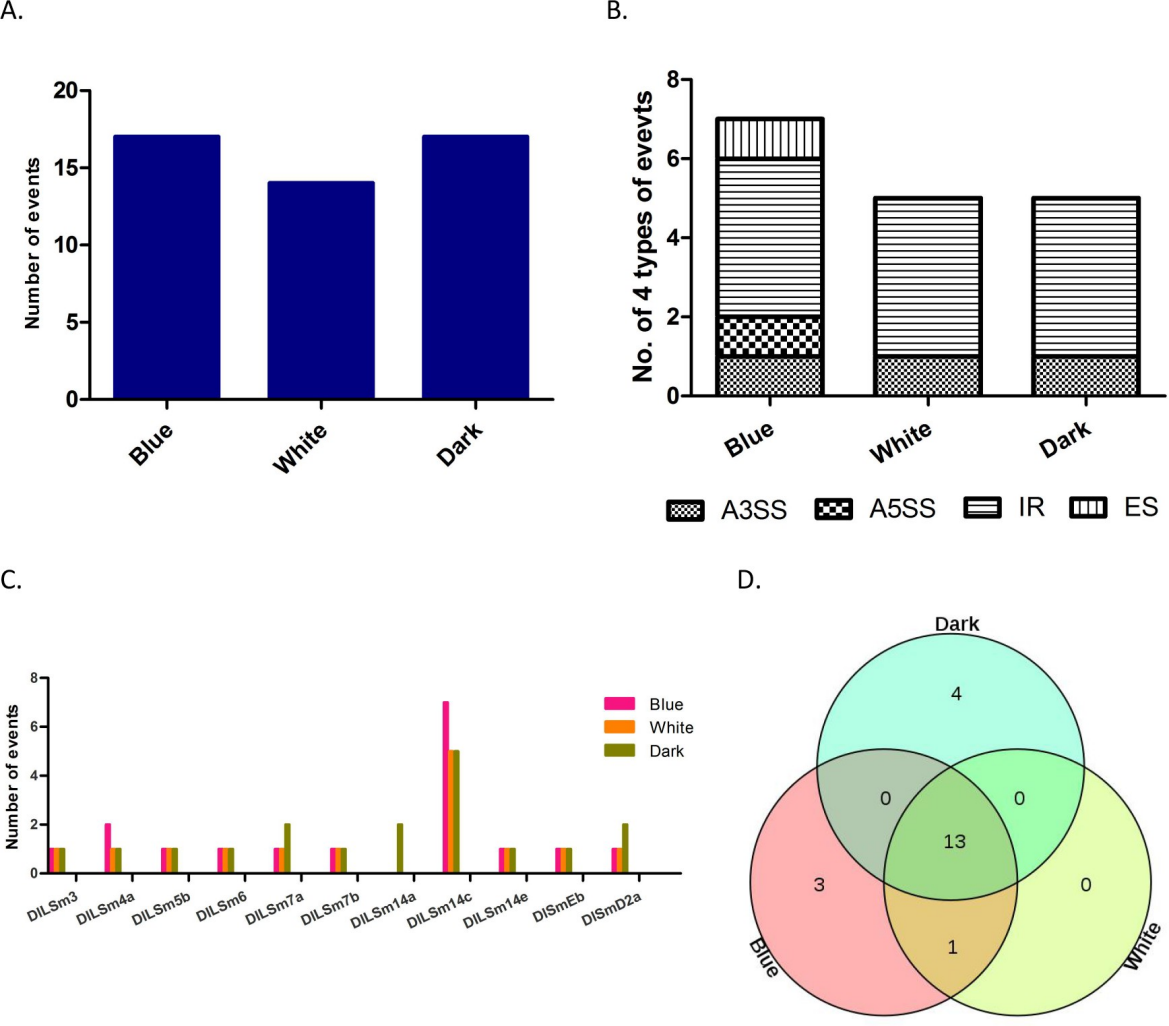
AS events of *DlSm* genes in longan EC treated with diffient light qualities. **A** The number of AS events in Longan EC under different light quality treatments. **B** Distribution of AS events in *DlLSm14c* under different light quality treatments. **C** Number of four types AS events of *DlSm* family members under different light quality treatments. **D** Specific splicing of *DlLSm14c* under different light quality treatments.

The growth and physiological changes of longan EC are known to be affected by light, and different light qualities have different effects on their growth[36, 42–44]. We analyzed the transcriptional data to explore the relationship between light quality and AS of *DlSm* genes. Longan ECs were treated with blue light and white light, and dark as the control (Fig 7A). We found the total numbers of AS events did not change after the blue light treatment, but decreased after the white light treatment compared with the control. We found that AS events occurred in ten *DlSm* genes under the blue and white light treatments, and in 11 *DlSm* genes under the dark treatment. The highest numbers of AS events occurred in *DlLSm14c* under the three treatments, and IR events occurred only in *DlLSm14c* under the three treatments (S4 Table). Under the blue light treatment, the A5SS and ES events of *DlLSm14c* were one more than under the other two treatments (Fig 7B). Compared with the dark treatment, four specific AS events were detected in the *DlSm* genes under the blue light treatment (Fig 7D) and one specific AS event was detected under the white light treatment. All the AS events that occurred under the white light treatment also occurred under the blue light treatment. There was no change in the numbers of AS events detected in the *DlSm* genes under blue light; however, AS events were more under blue light, and less under white light. These results suggest that blue light may affect the diversity of *DlSm* splicing more strongly than white light.

### The expression profiles of the *DlSm* genes in longan NECs, early SE, different tissues and organs of longan and ECs under different hormones and light quality treatments

**Fig 8:**
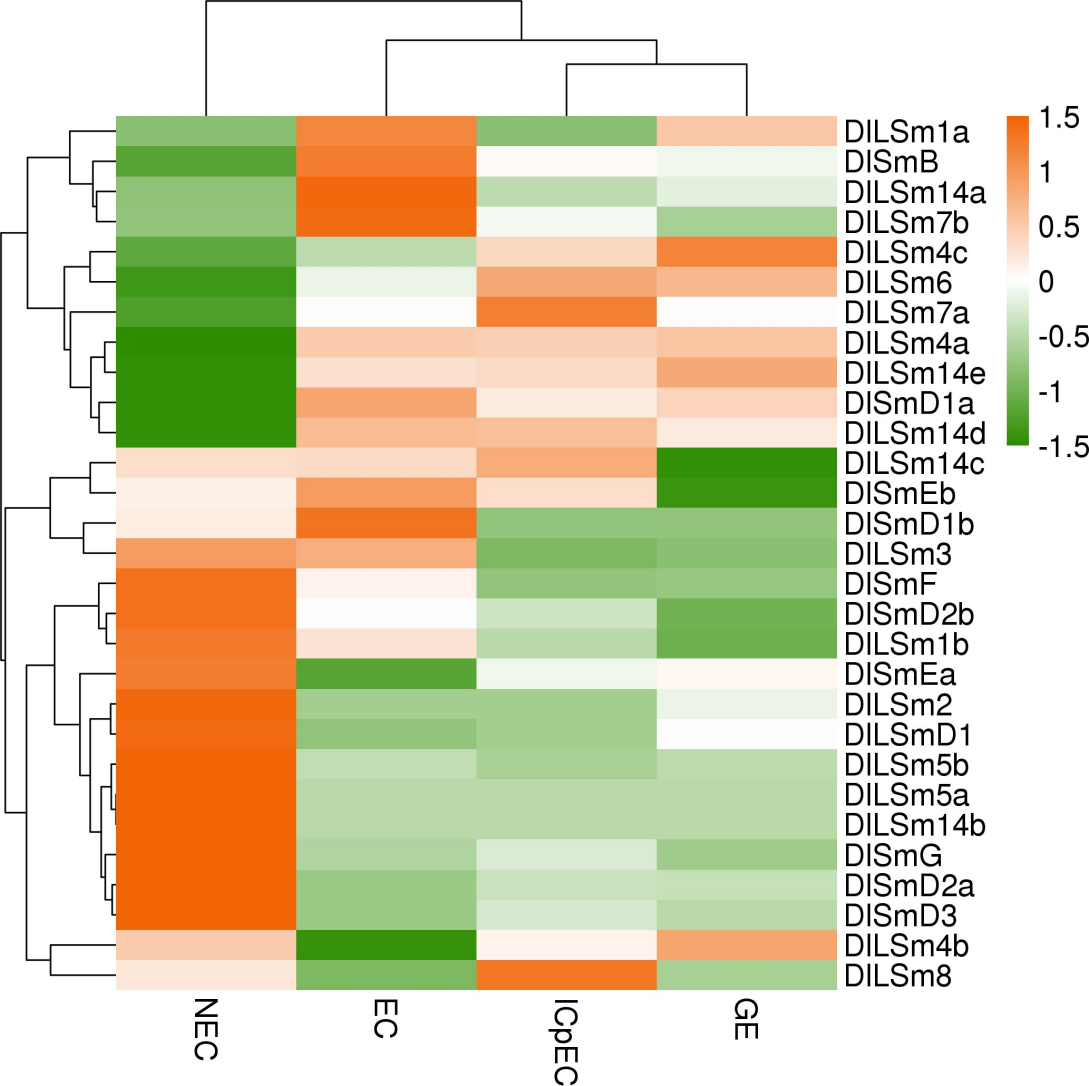
The FPKM of the *DlSm* family in NEC and the early SE stages of 'Honghezi' longan.

Expression profiling is used widely to understand gene function[45]. To investigate the expression profiles of the *DlSm* genes, we analyzed their FPKM values of *DlSm* genes in NECs, early SE (EC, ICpEC, GE) stages of 'Honghezi' cultivar SE and different tissues and organs of the 'Sijimi' cultivar were analyzed by cluster analysis. The results showed that in the NEC stage and the early SE stages (Fig 8; S5 Table), 12 *DlSm* genes (*DlSmF*, *DlSmD2b*, *DlLSm1b*, *DlSmEa*, *DlLSm2*, *DlLSmD1*, *DlLSm5b*, *DlLSm5a*, *DlLSm14b*, *DlSmG*, *DlSmD2a*, *DlSmD3*) were most highly expressed in the NEC, seven (*DlSmD1a*, *DlLSm1a*, *DlSmB*, *DlLSm14a*, *DlLSm7b*, *DlSmEb*, *DlSmD1b*) were most highly expressed in the EC, *DlLSm3* was most highly in the NEC and EC stages, *DlLSm8* was most highly in the ICpEC, and *DlLSm4c* was most highly expressed in the GE stage. Remarkably, some members of same phylogenetic group had similar expression patterns, such as *DlLSm5a* and *DlLSm5b*, which were highly expressed in NECs and lowly expressed in the EC, ICpEC, GE stages. The expression patterns of some group members were complementary, such as high expression of *DlLSm14a* in ECs and low expression in the other three stages, high expression of *DlLSm14b* in NECs and low expression in the other three stages, and high expression of *DlLSm14d* and *DlLSm14e* in the EC, ICpEC and GE stages, low expression in the NEC stage. The equally complementary groups were Group Ⅰ (*DlLSm4)* and the Group Ⅻ (*DlSmD1)*. The above members, which were highly expressed in the EC, ICpEC and GE stages, may play an important role in maintaining of SE in longan.

**Fig 9:**
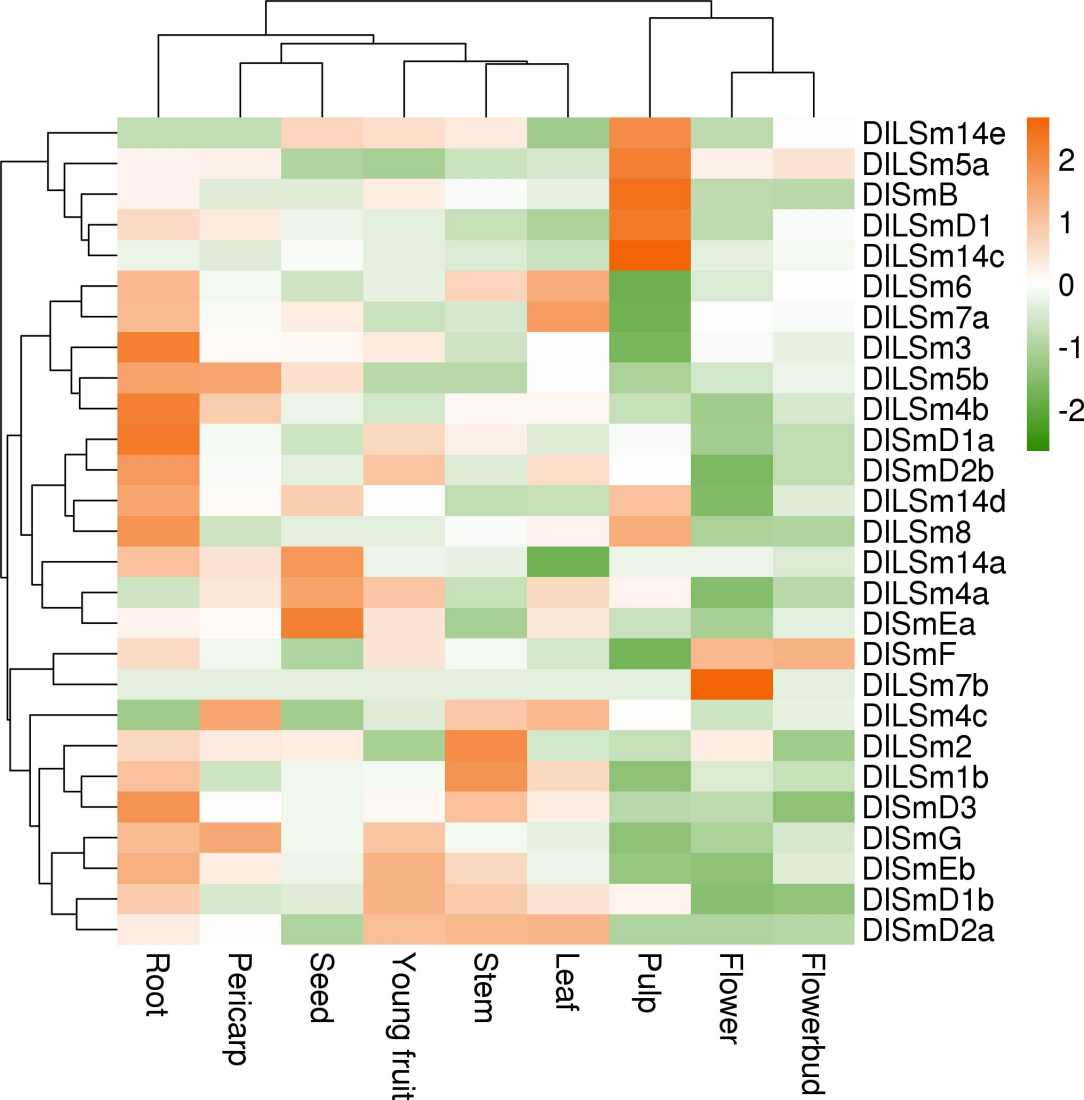
The FPKM of the *DlSm* family in different tissues and organs of 'Sijimi' longan.

Analysis of the FPKM values of the *DlSm* genes in different tissues and organs of longan (Fig 9; S6 Table) showed that *DlSm* genes were expressed in all the tissues tested, but their expression patterns were different. For example, *DlLSm7b* was most highly expressed in flower, and *DlLSm14e*, *DlLSm5a*, *DlSmB*, *DlLSmD1* and *DlLSm14c* were highly expressed in pulp, and *DlLSm3*, *DlLSm4b*, *DlSmD1a*, *DlSmD2b*, *DlLSm8*, and *DlSmD3* were significantly higher in roots than that in other tissues. In stem, *DlLSm2* and *DlLSm1b* were expressed higher than in other tissues, and *DlLSm14a*, *DlLSm4a*, and *DlSmEa* were highly expressed in seeds. The expression patterns of the *DlSm* genes in the nine tissues tested did not show any obvious similarity or complementary relationship.

**Fig 10:**
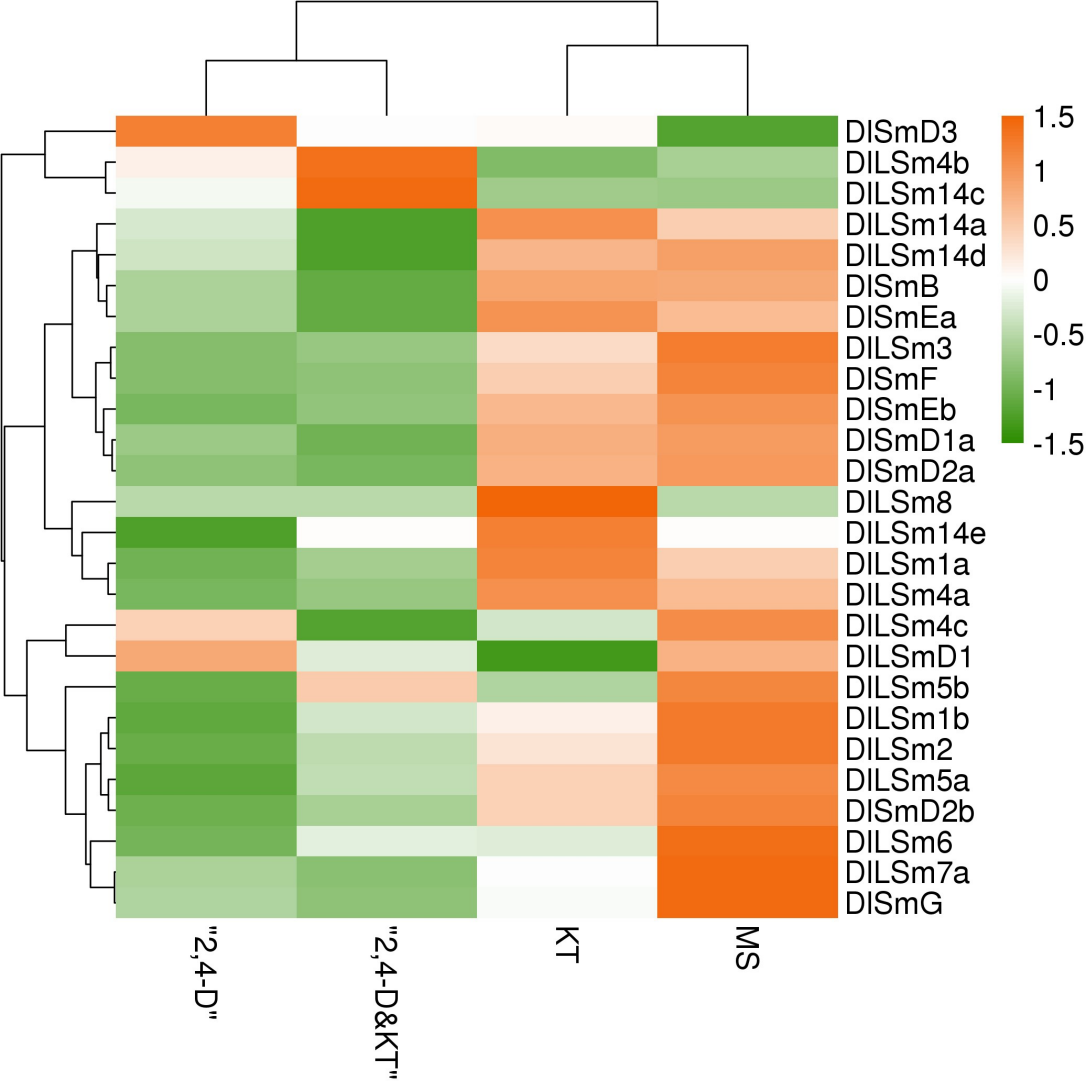
The FPKM values of the *DlSm* family in longan EC stage treated with different hormones.

Sm proteins are RNA binding proteins that play an important role in RNA metabolism, while plant growth regulators have an important affect the growth and induction of SE in longan. Until now, the effects of growth regulators on the *DlSm* genes involved in longan SE have not been reported. Therefore, we analyzed the FPKM values of the *DlSm* genes in longan EC treated with three different hormones (Fig 10; S7 Table). Under the 2,4-D, 2,4-D&KT, and KT treatments, the numbers of *DlSm* genes that were highly expressed decreased. Most of the *DlSm* genes had low expression under the 2,4-D, and 2,4-D&KT treatments, and *DlSmD3* was highly expressed only under the 2,4-D treatments and *DlLSm4b* and *DlLSm14c* were highly expressed only under the 2,4-D&KT treatment. However, more than 50% of the genes were highly expressed under the KT treatment, and *DlLSm14e* and *DlLSm8* were highly expressed only under this treatment. KT can induce the differentiation of ECs, so the highly expressed *DlSm* genes that were highly expressed under this treatment may promote the occurrence of RNA events related to differentiation.

**Fig 11:**
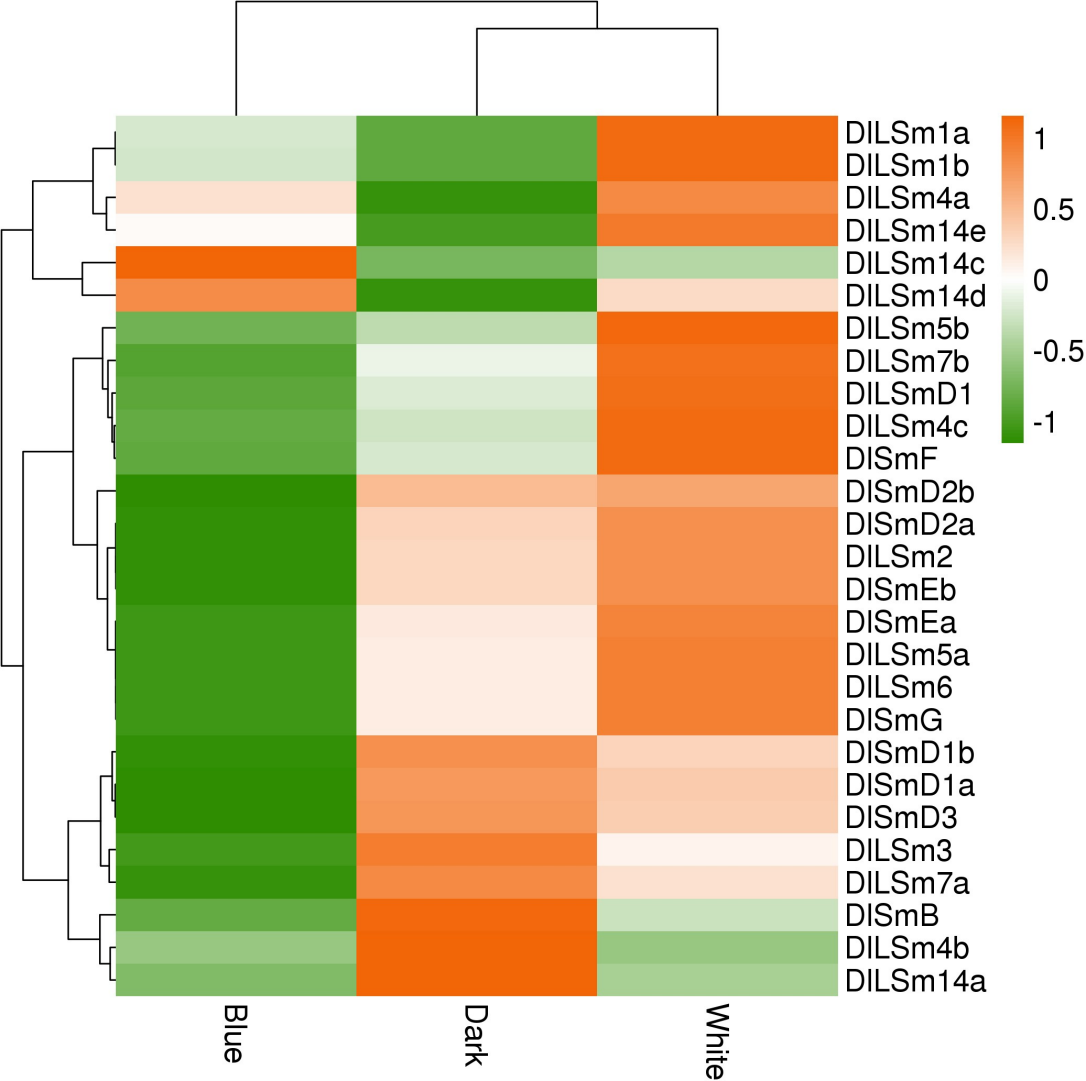
The FPKM of the *DlSm* family in longan EC stage treated with different light qualities.

We also analyzed the FPKM values of the *DlSm* genes in longan EC stage treated with different light qualities. We found that more *DlSm* genes were highly expressed under the white light treatment than under the dark treatment, and the genes with high expression under the white light treatment are usually low expressed under the dark treatment, whereas most of the *DlSm* genes showed low expression under the blue light treatment (Fig 11; S8 Table). Among them, *DlLSm14c* was highly expressed only under the blue light treatment, and *DlLSm1a*, *DlLSm1b*, and *DlLSm14e* were highly expressed only under the white light treatment, and *DlSmB*, *DlLSm4b*, and *DlLSm14a* showed low expression under both the white and blue light treatments. The above results showed that the expression of the *DlSm* genes were different under the different light quality treatments. In general, white light promotes the expression of the *DlSm* genes in longan ECs, whereas the blue light inhibited the expression of the *DlSm* genes in the ECs.

### Verification of the expression of the *DlSm* genes by qRT-PCR

**Fig 12:**
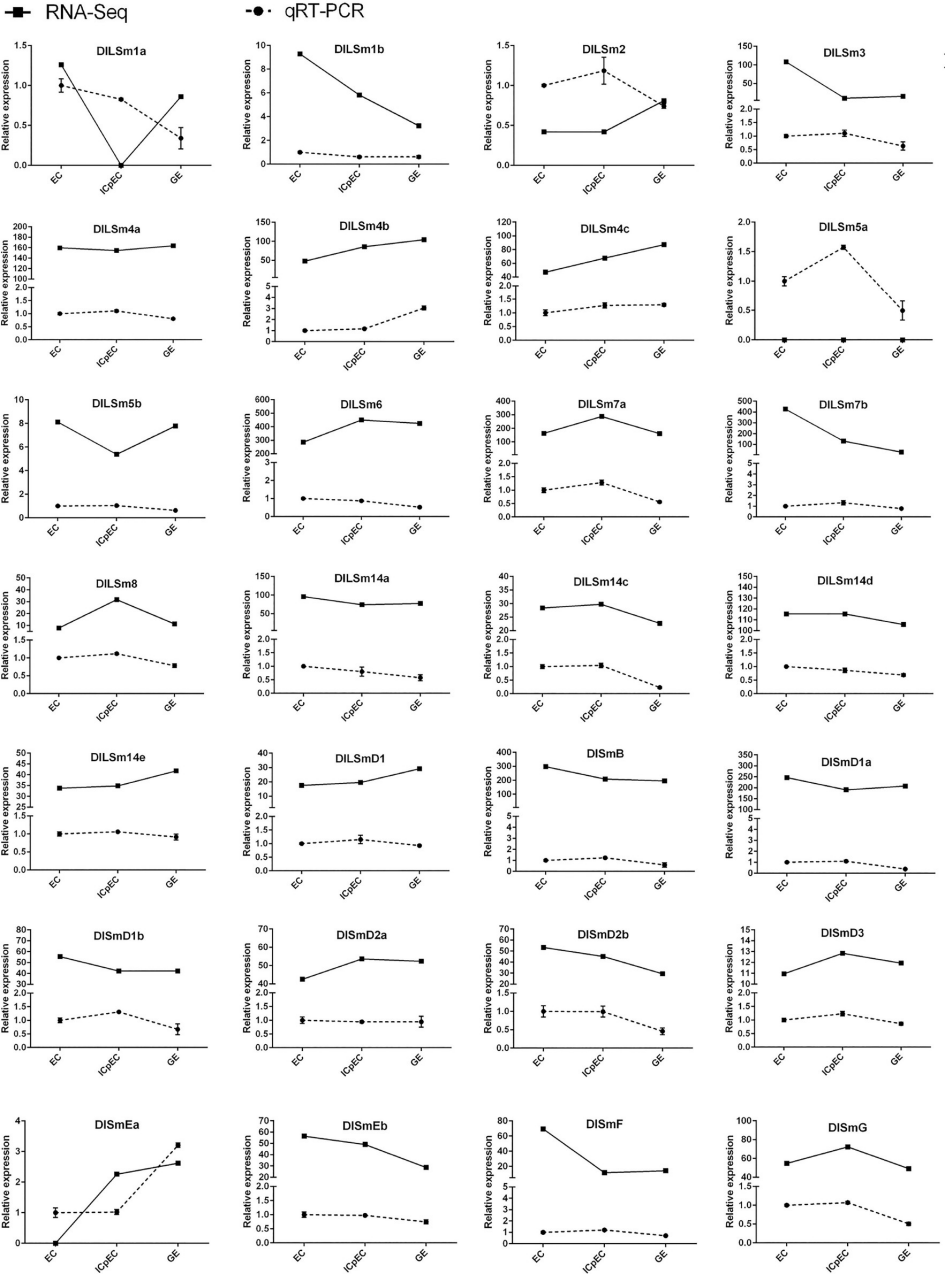
Comparison of expression trend of the FPKM and qRT-PCR. *EF-1α* was used as a reference gene to normalize the FPKM value and qRT-PCR expression data of *DlSm* genes. Active line section represented FPKM value; dotted line graph represented qRT-PCR results and vertical bars showed standard deviation.

In order to identify the expression of *DlSm* genes in the early stage of longan SE, we selected the 28 *DlSm* genes that were expressed in the early stage of SE in longan for verification by qRT-PCR(Fig 12). The results showed that the expression trends obtained from the of RNA-Seq data (FPKM values) and the qRT-PCR data at the three SE stages were the same for eight of the selected *DlSm* genes; however, the expression trends were opposite for some genes including *DlLSm1a*, *DlLSm2*, *DlLSm3*, and *DlSmEa*. These discrepancies may be related to differences in the batch of the material and the growth state that were used. The expression trends obtained from the RNA-Seq data and qRT-PCR data was consistent for 15 of the *DlSm* genes from the EC to ICpEC stages and from the ICpEC to GE stages, respectively. However, the expression trends of *DlLSm1a*, *DlLSm2*, *DlLSm4b*, *DlLSm5a*, *DlLSm14a*, *DlLSm14c*, *DlSmD2b*, *DlSmEa*, and *DlSmG* varied greatly from the EC to GE stages. The expression of 25 genes decreased from the EC to GE stages. These results showed that the expression of *DlSm* genes was affected by the growth and differentiation of somatic embryos, and their expression decreased as the increase of somatic embryo differentiation progressed.

## Discussion

### Longan Sm proteins are highly conserved and DlLSm14 protein may have functional diversity

We identified 29 *DlSm* genes of longan were identified in this study. Comparison of the deduced DlSm protein sequences with homologous Sm proteins from *Arabidopsis* showed that they contained the conserved LSm domain that is typical of the LSm protein superfamily, indicating that Sm proteins are highly conserved among different species. However, some domains varied among different members of the same phylogenetic group. For instance, DlSmB in Group XIII had one more DNA_pol3_delta2 superfamily domain than the two members of *Arabidopsis* proteins in this group, and the LSm14 proteins in Group IV had an extended C-terminal, and most had an FDF protein domain in the N-terminal. The FDF domain is a complete α-helical domain with multiple exposed hydrophilic rings rich in glycine and charged residues, a feature that is unique to LSm 13–16[46–48]. LSm14 protein has been shown to contain three highly conserved DFDF/FFD/TFG motif boxes, which not only are involved make it participate in RNA metabolism like the other LSm proteins, but are also involved in the regulation of the mitotic G2/M phase[48–50]. We also found the DFDF/FFD/TFG motif boxes in longan LSm14 protein(S1 Fig). These results suggested that the structures of the Sm proteins were highly conserved in structure, but the specific domains in some of the proteins may contribute to their functional diversity. However, in the present study we focused on the function and structure of the small Sm and LSm proteins with no more than about 150 residues. Little is known about the large LSm domain proteins that contain additional domains[48]. The results of FPKM analysis revealed a complementary relationship in the gene expression patterns among different members of the same phylogenetic subgroup, indicating that there may be complementarities in their functions. DlLSm14 proteins also had showed different expression patterns in the NEC stage and early stages of SE. These results led us to speculated that DlLSm14 protein may play an important role during SE of longan; however, this needs to be verified in future studies.

### The promoters of longan *Sm* genes contain a large number of *cis*-acting elements involved in response to abiotic stress, circadian rhythm, and organ development

The growth, development and metabolism of plants are affected by many factors. Cristian et al.[17] found that the function of the LSm2–8 complex in *Arabidopsis* was controlled by external signals, and the adaptability of plants to abiotic stress was regulated differently. The level of U6 snRNP, which is closely related to the complex, changed splicing activity according to the external environment. Cui et al. [18] through a comprehensive transcriptional group analysis of *Arabidopsis thaliana*, it was proved that the variable splicing event of stress response gene was induced by SAD1 gene encoding lsm5 protein under salt stress. Seo et al.[51] overexpressed *CCA1α* and *CCA1β* genes in *Arabidopsis thaliana* at a low temperature, which showed that the cold regulation of *CCA1* selective splicing increased the freezing tolerance of *Arabidopsis* and regulated the biological clock. AS of Sm genes can regulate plants’ biological clocks and their resistance to abiotic stress by splicing. However, until now the promoters of *Sm* genes have not been analyzed. We found that the promoters of *DlSm* genes contained a large number of physiological circadian rhythm control response elements as well as abiotic stress response elements such as hose related to mechanical injury, drought, low temperature, and anaerobic induction. In addition, 50% of the *DlSm* promoters contained protein metabolic response elements, which may play a role in maintaining the stability of protein metabolism.

The *DlSm* promoters also contained a large number of endosperm development response elements, but only *DlLSm14a*, *DlSmEa*, and *DlLSm4a* were found to be up-regulated in the seeds according to the FPKM values for the different longan tissues. SmB has been shown to be essential for stem cell regeneration, and LSm14 is related to mitosis of animal oocytes, and different mutations in SmD1a, SmD1b, SmD2a, SmD2b, and SmF were shown to kill yeast cells[49,52–54]. We found that promoters of *DlSmD2b* and *DLSmEa* responded to cell cycle regulation. The suitable concentration of 2,4-D was found to be beneficial for callus production and inhibition of proembryo formation[55], we found the promoters of *DlSmD1a*, *DlSmB*, *DlLSm14c*, and *DlLSm14e* contained auxin response elements and these genes showed different expression patterns at different somatic embryo growth stages. Further, *DlSmD1a*, *DlSmB*, *DlLSm14c*, and *DlLSm14e* contained elements related to meristem expression. Whether these proteins also have the unique functional roles remains to be further investigated.

### KT may affect the AS of *DlSm* genes and thus affect the differentiation of longan EC

Alternating the culture medium containing 2, 4-D as well as that containing 2,4-D、KT and AgNO_3_ was shown to be beneficial to the long-term maintenance of longan EC[31]. A suitable concentration of 2,4-D could maintain the embryogenic calli of longan, and also promoted ethylene and accelerated the senescence of longan EC, whereas KT promoted the growth of EC, resulting in somatic embryo differentiation, so alternating the cultures could control the negative effects of the two hormones[31, 41]. We found that compared with the MS treatment, the AS events in the longan EC decreased under the three hormone treatments, and the AS events in *DlLSm14c* were different under the different treatments. We also found that the number of AS events first decreased in the early stage of SE; therefore, we speculated that the production of the specific gene variants may be related to the differentiation of longan somatic embryos induced by KT and 2,4-D.

The comparative analysis of the expression profiles of *DlSm* genes in longan SE obtained from the RNA-Seq data (FPKM values) and qRT-PCR analysis showed that their expression was affected by the growth and differentiation of longan somatic embryos. The expression of most of the genes decreased from the EC to GE stages, which we speculated may be related to cell differentiation. We found that the expression of *DlLSm8* and *DlLSm14e* was specific under the KT treatment, that of *DlSmD3* was specific under the 2,4-D treatment, and the expression of *DlLSm4b* and *DlLSm14c* were specific under the 2,4-D&KT treatment. These results suggest that the genes that were specifically expressed under the different treatments may be related to the different effects of the hormones on longan SE. The interactions among genes are very complex and are influenced by many factors, so whether the AS events produced under different hormonal treatments affect the translation of the mRNAs needs to be further verified.

### Blue and white light may affect the growth of longan EC by affecting AS events of *DlSm* genes

All kinds of light matter have been shown to influence the morphology, organ differentiation, and biochemical index of plant cells[56]. Different photoquality can have different effects on different plant embryos[43,57–59]. We found that AS events of the *DlSm* genes decreased under the white light treatment. Compared with the dark treatment, the blue light treatment produced five specific AS events and the white light treatment produced one specific AS event. The white light promoted the expression levels of the *DlSm* genes, whereas the blue light inhibited their expression levels. Further, and the high expression of the *DlSm* genes under the white light treatment was significantly higher than their expression under the blue light treatment. *DlLSm14c* was expressed only in blue light and eight genes were expressed only in white light. Thus, the expression levels of the *DlSm* genes were promoted under the white light treatment and the AS events were reduced, whereas the expression levels of most of the genes were suppressed under the blue light treatment. These results, led us to speculated that blue light may increase the AS events in the *DlSm* genes, so their normal transcripts were not produced and their expression levels were suppressed. These two situations may trigger different resistance mechanisms in longan because of light quality stress. Our hypothesis about the effects of the two kinds of light quality on longan EC is consistent with that of Lin Xiulian[60], who found that blue, white, red, and green light were not conducive to the growth of longan EC. However, Zeng Lilan[61] showed that blue light and green light were more beneficial to the growth of longan EC than white and red light. Our results are different from those of the previous studies, which may be related to the composition of culture medium and the wavelength used for the light quality treatments.

## Conclusions

In this study, we identified 29 *Sm* genes in 17 phylogenetic groups in longan. Most *DlSm* genes are respond to various plant hormones, such as methyl jasmonate, salicylic acid, abscisic acid, ethylene, and auxin, and some genes may regulate the development of the early SE and the tissues(seed, flower, pulp and root) of longan. The AS events were different in the four development stages, and may be related to the differentiation of longan somatic embryos. The expression profiles of the *DlSm* genes indicated their possible functional divergence. Notably, the numbers of AS events of *DlLSm14* were significantly more than in the other genes both under the different hormone treatment and different photoquality treatments. The results show that the *DlSm* genes may play an important roles in SE of longan. The analysis and results reported here will help to identify candidate genes for further functional studies.

## Supporting information

S1 Fig Domains of DlLSm14 proteins. (PNG)

S1 Table The primers of qRT-PCR. (DOCX)

S2 Table Statistic of total AS events called from all samples in longan NEC stage and early SE stages. (XLSX)

S3 Table Statistic of total AS events called from all samples in longan EC treated with different hormones. (XLSX)

S4 Table Statistic of total AS events called from all samples in longan EC treated with different light quality. (XLSX)

S5 Table The FPKM value of NEC and early SE of 'Honghezi' cultivar. (XLSX)

S6 Table The FPKM value of the nine organs of 'Sijimi' cultivar. (XLSX)

S7 Table The FPKM value of EC under the different hormone treatments. (XLSX)

S8 Table The FPKM value of EC under the different light quality treatments. (XLSX)
