# Supplementary figures and images for: Genome-wide identification and expression analyses of Sm genes reveal their involvement in early somatic embryogenesis in Dimocarpus longan Lour
Source: PLoS One. 2020 Apr 3;15(4):e0230795. doi: 10.1371/journal.pone.0230795 (PMC7122786; doi:10.1371/journal.pone.0230795)

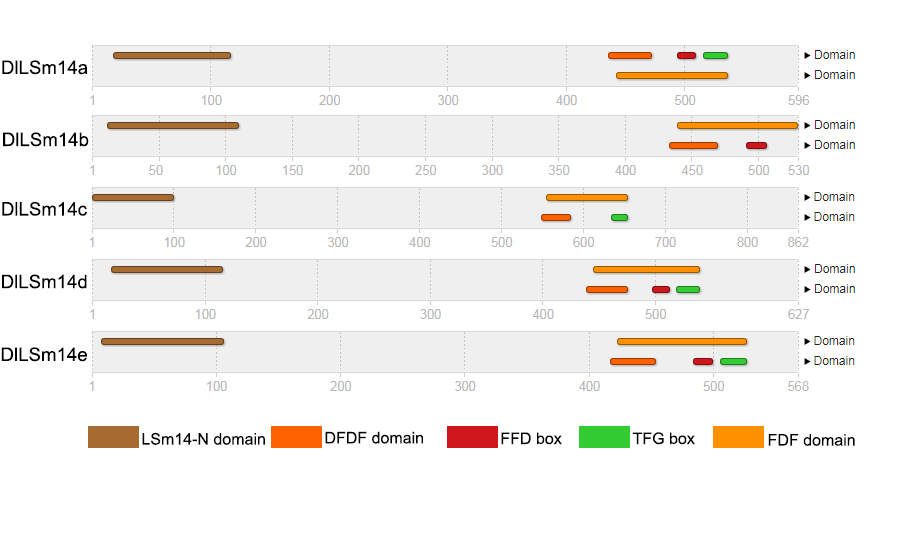

Supplement: S1 Fig — (PNG) [file pone.0230795.s001.png]
