# Supplementary material for: Genome-wide identification and expression analyses of Sm genes reveal their involvement in early somatic embryogenesis in Dimocarpus longan Lour
Source: PLoS One. 2020 Apr 3;15(4):e0230795. doi: 10.1371/journal.pone.0230795 (PMC7122786; doi:10.1371/journal.pone.0230795)
Supplement: S1 Table — (DOCX) [file pone.0230795.s002.docx]

**Table S1 The primers of qRT-PCR**

| **Primer name** | **Primer sequences(5’-3’)** |
| --- | --- |
| Dlo_014464.1-Forward | CCGCTGTCTGTATTGATGATG |
| Dlo_014464.1-Reverse | TTTTCTTGCCTTTCCCAGTC |
| Dlo_009060.1-Forward | AACTGCCGGAACAACAAGA |
| Dlo_009060.1-Reverse | CACCTTTGCTCCTCCCCTA |
| Dlo_012057.1_Forward | GCCAACAATCCTTCACAGC |
| Dlo_012057.1_Reverse | CAGGAACCAGAATCGCAAT |
| Dlo_023124.1_Forward | TGAGCGAATAATTGTTGGGG |
| Dlo_023124.1_Reverse | GCGGTAAGAAGCTCTCCTCA |
| Dlo_023126.1_Forward | TGAGCGAATAATTGTTGGGG |
| Dlo_023126.1_Reverse | ATCCCTTCTGAGCCCTTTTT |
| Dlo_021780.1_Forward | CAAGTGGGGAATGGAGTACA |
| Dlo_021780.1_Reverse | TTCATCCTCCGGTACACCA |
| Dlo_022245.1_Forward | TCCTCAAATCAATCCGTGG |
| Dlo_022245.1_Reverse | CCCCGTCTGCTAATGTCCT |
| Dlo_032946.1_Forward | CCTCAAATCAATCCGTGGGC |
| Dlo_032946.1_Reverse | CCTCAAATCAATCCGTGGGC |
| Dlo_026784.1_Forward | CCTGTGATGCTCTCGATGG |
| Dlo_026784.1_Reverse | GGCCATATCCACGACCTCT |
| Dlo_011575.1_Forward | GAAGTATGGGGTCATCTCGG |
| Dlo_011575.1_Reverse | GATCCACCGTCAAGAGCAT |
| Dlo_009662.1_Forward | CCAGCCTCCAGATCTCAAGA |
| Dlo_009662.1_Reverse | TTCAAGAGCCTCGACCGTA |
| Dlo_009273.1_Forward | GCATCTATGTCAAGCTCCGA |
| Dlo_009273.1_Reverse | CCCCCATCATCAATCTCTACA |
| Dlo_020209.1_Forward | GGAGTGGGGAGTTCAGGTG |
| Dlo_020209.1_Reverse | TGTCAATGCAGTGGAAGGTG |
| Dlo_024845.1_Forward | GGCACTCTCCACTCCGTT |
| Dlo_024845.1_Reverse | CCCTAGTGTTCTCGAGCTTGA |
| Dlo_033912.1_Forward | GCTCCGCCAATGACTTATC |
| Dlo_033912.1_Reverse | CTCACTCCAAATGCAGCAG |
| Dlo_038407.1_Forward | ACAGAGGAAGCATGGTCGAG |
| Dlo_038407.1_Reverse | AAGACGCTTGAACATCGGAG |
| Dlo_024823.1_Forward | GATCTCCAGCTGAAGACCAACT |
| Dlo_024823.1_Reverse | CGAGCAGGGTCGGACTTA |
| Dlo_031697.1_Forward | CCTCAGTGTGCGGGGTAA |
| Dlo_031697.1_Reverse | GACCACGTCCCACTCCAG |
| Dlo_014195.1_Forward | TGGAGATAGGTTTTGGAGGATG |
| Dlo_014195.1_Reverse | CATCTAGTCCACGCCCGA |
| Dlo_021351.1_Forward | CCCTTAGTACCTCCGTCATCCA |
| Dlo_021351.1_Reverse | AGATCGTAGGCCACCTGG |
| Dlo_031761.1_Forward | AGTCGTTCAGATCGTAGGCC |
| Dlo_031761.1_Reverse | CCACCTGCACCCTTAGTACC |
| Dlo_034364.1_Forward | CGGTAGACAAGTAACAGGGAC |
| Dlo_034364.1_Reverse | GGGTTGGCAATCTCATCTGT |
| Dlo_028712.1_Forward | GCAGACAAGTGACAGGGACTC |
| Dlo_028712.1_Reverse | TGGGTGAAAGGGTTGGC |
| Dlo_036673.1_Forward | TCATGAGCGTGTCTACTCCAC |
| Dlo_036673.1_Reverse | GACAGGCTTGAGGGGATG |
| Dlo_029021.1_Forward | TCTCCGCCGTTAACATCAG |
| Dlo_029021.1_Reverse | TGGATTCAACAGACCTGCC |
| Dlo_006168.1_Forward | TACCCCCCTTGGTTAATGAC |
| Dlo_006168.1_Reverse | GTTGGACGGGCAGTTGAT |
| Dlo_019122.2_Forward | CAGCGGATTATGACTCAACC |
| Dlo_019122.2_Reverse | GCCCGTGTTCATCATCAG |
| Dlo_015839.1_Forward | CGGCGGTTTTGATGAGTAC |
| Dlo_015839.1_Reverse | GGCGGATCAGAGTTATGTTG |
